# Supplementary material for: An Assessment of Vegetation Changes in the Three-River Headwaters Region, China: Integrating NDVI and Its Spatial Heterogeneity
Source: Plants (Basel). 2024 Oct 8;13(19):2814. doi: 10.3390/plants13192814 (PMC11479222; doi:10.3390/plants13192814)
Supplement: Supplementary file 1 [file plants-13-02814-s001.zip › plants-3201234-supplementary.pdf]

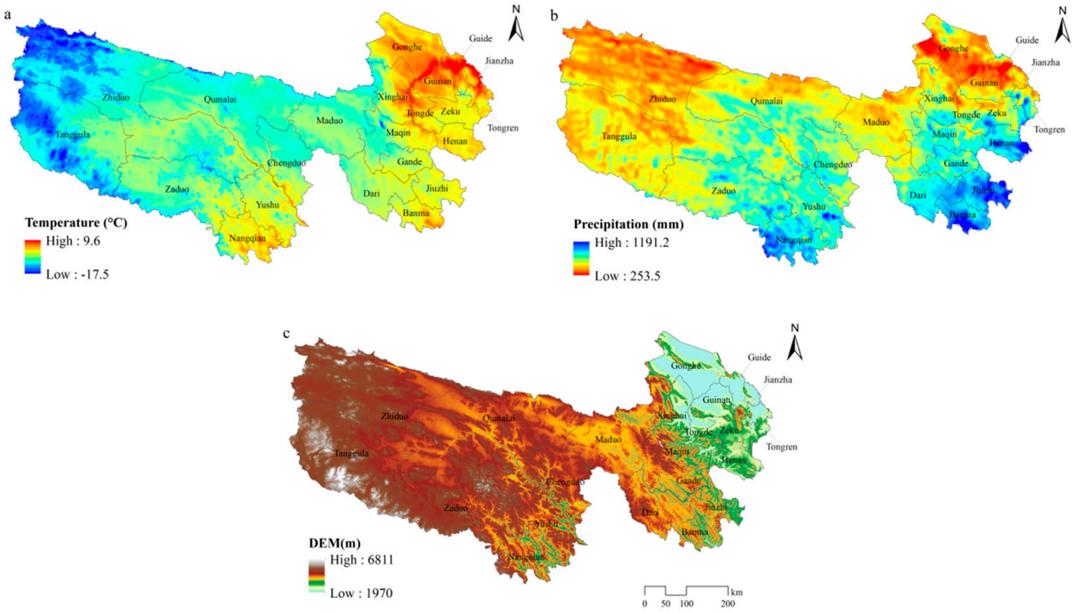

Figure S1. Annual average temperature and precipitation, elevation of the study area

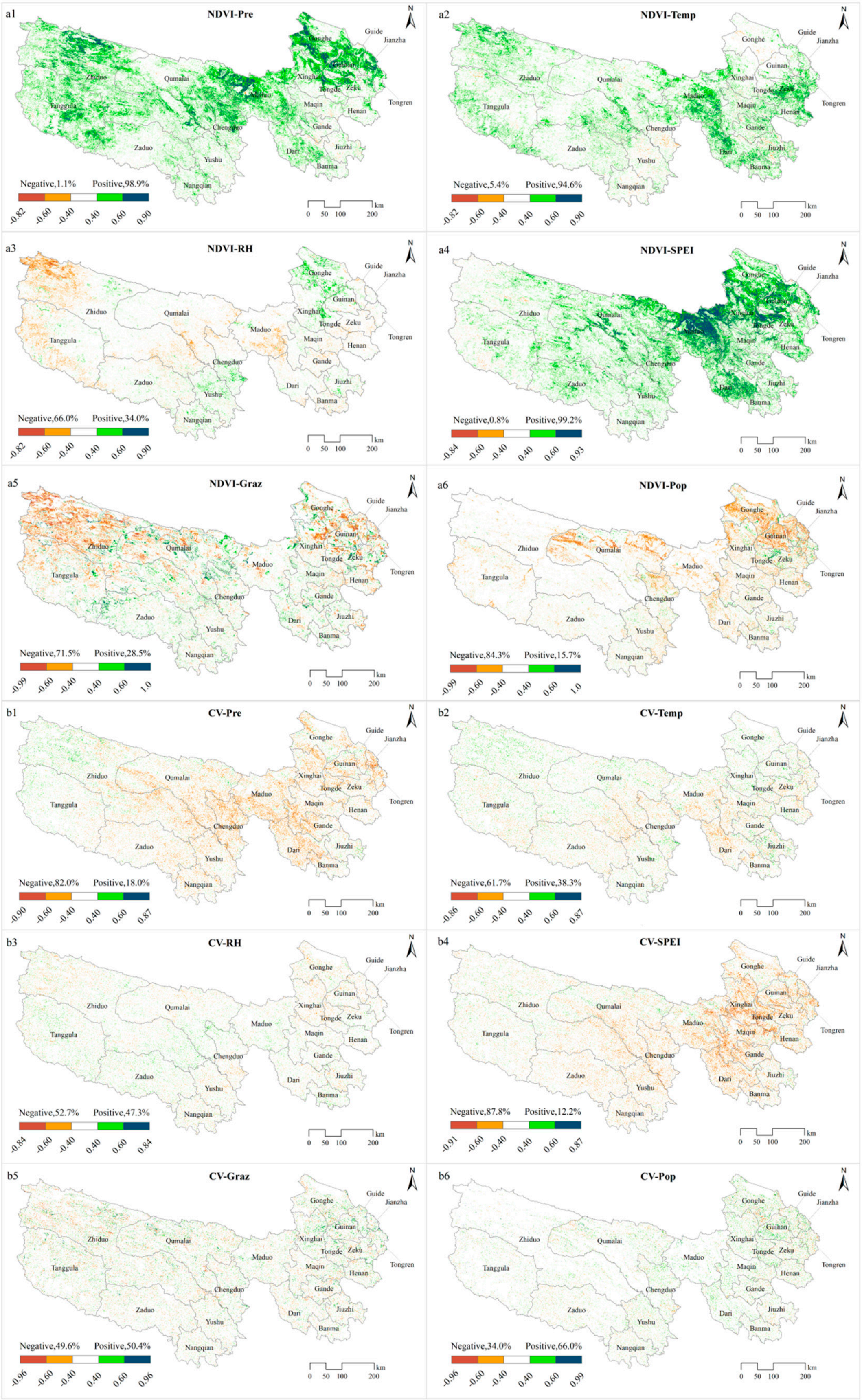

Figure S2. Correlation coefficients of different factors with NDVI (a1-a6) and its spatial heterogeneity (CV for short) (b1-b6) ($p<0.05$)

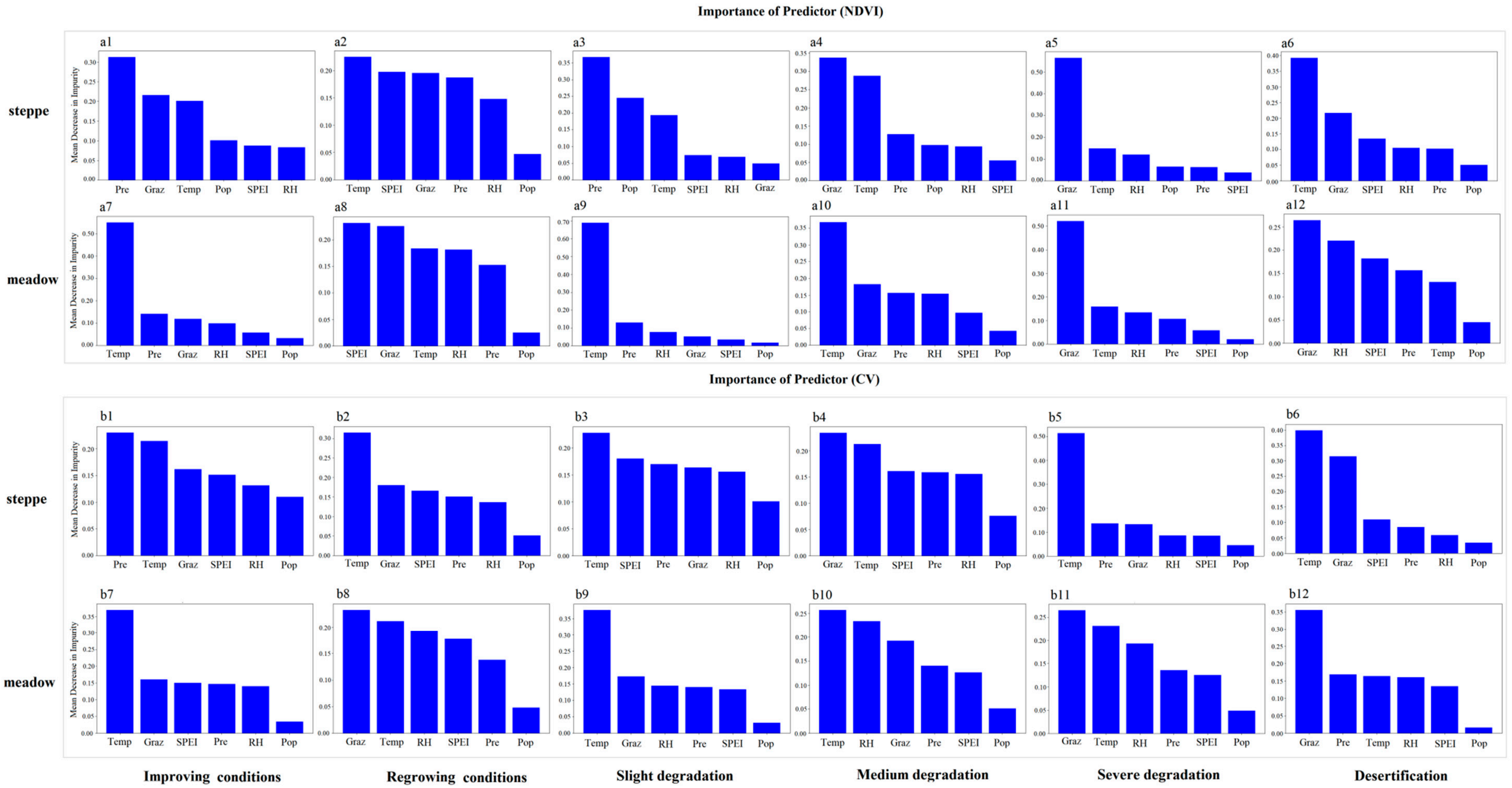

Figure S3. Feature importance of different driving factors to NDVI (a1-a12) and its spatial heterogeneity (CV for short) (b1-b12) (Numbers 1-6 and 7-12 represent six types of vegetation changes areas in steppe and meadow, respectively)

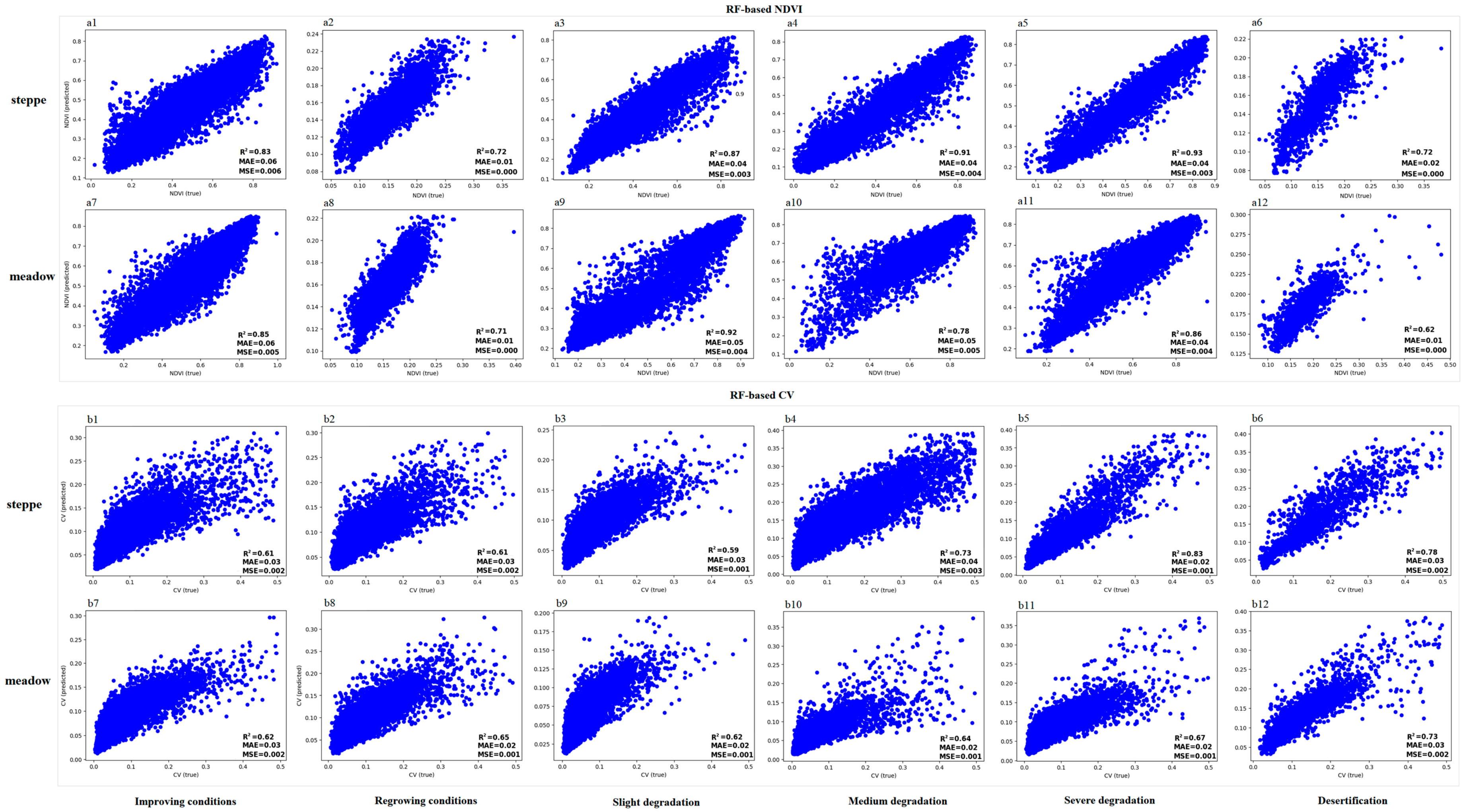

Figure S4. Scatter plots of predicted versus actual values of NDVI (a1-a12) and its spatial heterogeneity (CV for short) (b1-b12) (Numbers 1-6 and 7-12 represent six types of vegetation changes areas in steppe and meadow, respectively)
